# Statistical Medicine or Numerical Analysis Applied to the Investigation of Morbid Actions: A Lecture, Delivered, by Request, to the Medical Class of the University of Louisville, January 17, 1848

**Published:** 1848-03

**Authors:** Samuel A. Cartwright

**Affiliations:** Natchez, Miss.


### THE WESTERN JOURNAL OF MEDICINE AND SURGERY.

MARCH, 1848.

Art. I.—*Statistical Medicine or Numerical Analysis applied to the investigation of Morbid Actions:* A Lecture, delivered, by request, to the Medical Class of the University of Louisville, January 17th, 1848. By Samuel A. Cartwright, M.D., of Natchez, Miss.

Gentlemen—

I am about to say something on *Statistical Medicine*, or *Political Arithmetic* as it is sometimes called. Some have named it the *Mathematics of Medical Science;* others, *Numerical Analysis*, applied to the investigation of morbid actions. It is defined to be "the application of numbers to the elucidation of the natural history of man in health and disease." The success of numerical analysis, applied to morbid actions, in establishing the doctrine of averages with mathematical certainty, led to the application of the same kind of analysis to governmental objects and gave to political economy the title of a science. The new science of political economy is, therefore, the offspring of statistical medicine. It is only by extending observations over large masses of people and through a series of years, that the correct and certain data are obtained on which the science of medical statistics is founded. It tests the truth of theories. All medical opinions or practice founded thereon must stand or fall when touched by it. It goes to the very bottom of the well and discloses truth, which often lies too deeply hid to be uncovered by the labor of any one individual practitioner of medicine. Its calculations are founded on multiplied elements, forming the data for the induction of important principles. The numerical method is, in reality, the Baconian philosophy applied to medicine, and reduces it, very nearly, to the exact sciences.

Gentlemen—There must always be diseases and structural derangements *incurable* as a natural incident of humanity. Otherwise man would not be mortal. On the other hand, there must ever be diseases and functional derangements of the organs of the human body readily *curable* by the inherent energies of the constitution. Otherwise all that portion of mankind, deprived of the benefits of medical science, would be threatened with total extinction. The incurable diseases on the one hand, and those which are cured by the efforts of nature on the other, constitute the foundation of scepticism in medicine. Nay, more; it is the ground-work on which empiricism has built its strongest batteries against the regular medical profession. When physicians fail to cure incurable diseases or fail to raise up the patient after he has fallen into an incurable condition, they are mocked by sceptics and assailed with great virulence by empirics.

Again. When patients rise from a bed of sickness by the strength of their constitutions, in despite of the improper interference of quackery, they are published in the newspapers, often by name, and pointed out to the public as living witnesses of wonderful cures performed by the advertised nostrums, and as most fortunate men, women, and children who have escaped the hands of the regular doctors. Whilst these living monuments of the power of the *vis medicatrix*

*naturæ* to cure diseases, in despite of the very nostrums, (which have got the credit for the cure,) are marshalled to uphold quackery in its unjust pretensions to having made the cures which nature made, the marble monuments of those who have died with incurable diseases under the hands of the physicians, are pointed at as proofs of the inefficacy, nay of the prejudicial influence of the regular remedies of the materia medica as prescribed by the medical faculty. No wonder, therefore, that public confidence should be often shaken in physicians, and that they should sometimes falter and be thrown into jarring discord with one another under the heavy cross-fire which scepticism and empiricism always direct against them, when incurable diseases happen to fall in the line of their practice. While it is admitted that there are two classes of affection—one which will prove fatal under any treatment, and another which will get well under almost any kind of treatment not absolutely poisonous or destructive, there is certainly a very large class of diseases, lying between the two extremes, which can be greatly benefitted by judicious treatment. The total annual mortality, therefore, in a series of years and among large masses of people must be less, when science governs the treatment, than when ignorance governs. The mortality must be greater, in proportion to the *greater* amount of quackery, and *less* in proportion to its decrease. Statistical medicine proves that this is the case.

In morals, as in medicine, we find three classes, very similar to the above. In morals, we find a class of reprobates, answering to the class of incurable diseases in medicine, whom all the clergy and all the religious instruction of the most pious persons cannot reform. On the other extreme, there is another class so amiable, kind, and benevolent, as scarcely to require moral lectures, the promptings of the clergy, or even religion itself to make them dutiful citizens and good neighbors; they are even pointed to by unbelievers as proofs of the inutility of orthodoxy in religion, because such persons generally make good citizens in despite of heterodoxy in religion, or without any religion at all. But between these two extremes there is a very large class of persons who are greatly benefitted by moral and religious teaching. Infidelity has denied this position and called in question the utility of the clerical profession, on very similar grounds to those occupied by scepticism and quackery in calling into question the utility of science in medicine. The clergy have utterly demolished this strong hold of infidelity by proving, through the means of unquestionable statistical truths, that notwithstanding the fact, that there is a class of persons so hardened and reprobate as to be uninfluenced by the moral and religious doctrines of the Bible, and another class so good, as to make peaceable, quiet, and industrious citizens, although deprived of gospel privileges, that nevertheless, the class of individuals, lying between these two extremes, is so large that moral teaching and religious instruction have a most wonderful effect in diminishing the amount of vice and immorality among every people who enjoy such advantages.—Statistical truths prove, that a people, deprived of moral and religious instruction or having only that which the ignorant and bigoted can give, are invariably more depraved, immoral and vicious than those who have the benefit of an enlightened, upright, and industrious clergy; and that the people enjoying such advantages, make the best citizens, are better able to govern themselves and to govern the state, and run in the line of their duties, by moral influences, without being pushed therein at the point of the bayonet. The numerical method, so useful in demonstrating the evils of a want of religious and moral teachers among any people, and the evils of quackery in religion and morals, will be found no less useful in demonstrating the evils of a want of scientific physicians, and the evils of ignorant pretenders in medicine. With religious statistics I have nothing to do, being out of my pathway, which leads into the wide field of medical statistics in search of facts, leading to truths, so plain and convincing, as to enable every one, learned or not learned, to judge impartially, which of the two, science or empiricism, is entitled to the patronage and support of the public. A few facts often lead to error; whereas all the facts taken collectively always lead to truth. An erroneous opinion, unfavorable to our profession, is often formed from a few facts, indicating a want of success in a limited number of cases, or in some neighborhood of limited extent, or during some short period of time; whereas all the facts, in regard to the practice of physicians, drawn from all quarters and through long periods of time, would at once correct that erroneous opinion

Before going farther, it may be necessary to define what is meant by the science of medicine in contradistinction to empiricism. It consists of two things—science and opinion. True science treats of things known—opinion of things unknown. In other words, medicine as a science, consists of fact and theory—principle and precept. The two must be taken together to make the regular physician, properly so called. Mere facts without being generalized or connected together by reason or theory, are often profitless, or mere stumbling blocks. Whereas theory or opinion, not founded on facts and supported by them in all its parts, is the worst species of empiricism when reduced to practice. The medical principles and precepts, taught at schools, ought not to be mistaken as all the requisites to make a scientific physician. They are only the seed of true science. Labor, experience, and observation should be superadded to make the seed grow and mature into good fruit. Natural talents, a good preliminary education, an early beginning, a love of labor, sound moral principles, and economy of time, are all more or less necessary to constitute the useful physician. The duties of all regular physicians do not consist in wasting their time in investigations, disputes, or bickerings on matters of opinion of little or no practical importance, but in union with one another in the grand and glorious work of preventing and curing diseases, and in relieving pain and human suffering of all kinds. The treatment of diseases consists in doing certain things, and in not doing certain things. Science gives much light, directing what ought to be done or what ought not to be done, but it does not always give as much as is desirable. Hence the ablest physicians have often to proceed cautiously and to feel their way along, avoiding the timidity of weakness and the rashness of ignorance—contenting themselves in humbly learning useful lessons at the bedside, and not in irritating discussions of subjects without utility. In proportion as medical men approach these requisites, (which are not mine, but Hippocrates's the Father of Medicine,) so much the plainer will statistical medicine prove the results of their practice to be more successful than any form or species of empiricism whatever. The great object of medicine being to prevent and cure disease, it necessarily follows that if diseases be prevented, or cured, population will increase rather than diminish. The increase or diminution of population, other things being equal, will go very far to answer the question of the relative merits or demerits of the regular and empirical practice.

Numerical analysis, applied to the subject, discloses the important truth, that all that portion of the human race, residing in those countries or localities which have no scientific physicians, is either decreasing or at any rate not iucreasing to any extent in number, and that the annual ratio of mortality is very great. Whereas, all those in any and every country, who take the advice and counsel of the regular medical profession, are increasing instead of diminishing in numbers, and the annual ratio of mortality is very small, in comparison to that of those who put their trust in quackery. Our Indians are daily disappearing. They trust their lives, when sick, to ignorant advisers, not having any regular physicians among them. If Indian quackery was as successful in curing diseases, as the science of the civilized man, surely the mortality would not be so great among them as to threaten the total extinction of the race. The white man has occupied this continent but a short time comparatively, yet he has grown and multiplied and become much greater in numerical strength than the aboriginal inhabitants ever obtained previously to its discovery. The white man brought with him scientific medicine, which the Indian never had. If Indian vegetable specifics and Indian doctors cannot save the Indian race from extinction, in what can they be expected to profit the white man? While the Indian race is decreasing rapidly under the influence of empiricism, the African race, in our southern States, is daily growing in numbers under the salutary influence of science, as practiced by our regular physicians. The African tribes, though most prolific, do not increase in the aggregate population in their native climate. Unprotected by any healing art, the deaths keep pace with the births. But as soon as they gain the protection of science, the mortality is diminished and population progresses. The people of African origin, in our free States, do not increase in population, because there is no established authority to make them take physic when they are sick, being by nature so much opposed to it, as seldom to take it unless compelled to do so by the strong hand of power. As soon as the regular Spanish physicians were driven out of Mexico, that country immediately ceased to increase in population. Quackery reigns supreme in Mexico, and is striving hard to get the ascendency in the United States, not by force of arms, but by degrading the medical profession, and driving men of talent in disgust from it to other pursuits for a livelihood. Throughout all Europe, statistical medicine proves that the mortality among that portion of the population who, from ignorance, prejudice, and the influence of empirics, do not avail themselves of the life-giving powers of the medical science, is about twice as great as among that other portion of the population who trust their lives to the regular physicians, and take no other medicine than the well known articles of the materia medica.

In Paris, during the epidemic cholera, those ignorant and deluded people who, so far from calling on the regular physicians for aid, most uncharitably believed that they had caused the cholera to get business, suffered incomparably more from the disease than those wiser and more discreet persons, who followed the advice of skilful physicians. The same thing occurred throughout Europe, Asia, and America; the ignorant and the prejudiced in flying to quackery for relief, found to their sorrow, that its performances did not come up to its promises. Jealousy and prejudice against the medical profession have always been marks of broad vulgarity and ignorance.

The makers and venders of nostrums and patent medicines have opened an extensive market for their commodities by fostering that natural jealousy and prejudice of the ignorant against the medical profession, and by representing the regular articles of the materia medica as most noxious or poisonous.

Physicians have nearly exterminated the small-pox from every part of the world where medicine is cultivated as a science, as also that other most formidable disease, the scurvy, which threatened, at one time, to destroy all commerce between nations, and would have robbed Great Britain of her colonial possessions and destroyed her navy, if science had not come to her aid and pointed out the means to cure and prevent it among sailors and marines. Although they have made vast strides in preventing or curing numerous other afflictions, which beset the path of humanity, yet owing to the artifices of the proprietors of nostrums and quack remedies, the ignorant and the vulgar, throughout the world, are made to look upon the regular medical profession with distrust and suspicion; and ignorant, but often good meaning men, are encouraged to set up opposition systems. The effect of all this is to degrade the profession, rendering it scarcely worth the attention of either physicians or empirics to follow it for a livelihood, but to enrich the artful impostors in our large cities and in Europe, who are the fabricators and proprietors of patent medicines and nostrums. The amount paid to these impostors by the people of the Mississippi Valley, more than doubles the amount of sales of the regular articles of the materia medica in the Valley. In proof of this, as I am informed by Professor Drake, it has been found impracticable to prevent our western apothecaries from acting as agents or subagents for the proprietors of patent medicines, since such agency, owing to the vast amount sold, brings in greater gains, at twenty per cent., than do the regular articles of the materia medica at a higher per cent. profit, by reason of the smaller amount vended.

Time will not permit me to go into statistical details and give facts and figures already filling many volumes. Every physician can look over them at his leisure in his library. They are as dry and uninteresting in themselves as the details of meteorological observations, until breathed upon by the animating breath of generalization. They then assume a form and shape terrible to the sight of all impostors in medicine. Medical statistics form a striking illustration of the inutility of mere masses of fact, piled one upon another, without order, system, or arrangement. They profit nothing, they tell nothing, until galvanized into life by reason, theory, or generalization. If physicians everywhere would take the trouble to animate them and bid them speak, they would tell a tale, which would elevate the regular profession high in public estimation, and sink quackery so low that it would die out for want of patronage.

Medical men have been so remiss in awakening into life the dead statistical matter, which for ages has laid dormant in their libraries, that it may truly be said, that statistical medicine has done the profession very little good, in consequence of its not having been animated and interrogated on subjects exclusively medical. It has been called into life and interrogated on other subjects, and its answers have been wonderful, and no less true than wonderful. By its answers it has proved itself to be a science in the strict meaning of the term. By it, as by all true sciences, the dark future can be penetrated in a greater or less degree, and coming events, hid from vulgar eyes in the womb of time, can be predicted with almost absolute certainty. Although no one knows or can know, the time when any one individual in health will die a natural death, yet statistical medicine can foretell, with almost positive certainty, when a thousand or ten thousand individuals, in health, will die a natural death. By virtue of this prescience or foreknowledge, disclosed by the numerical method, the various life-insurance companies calculate the amount of profit to be derived from their policies of insurance.

Napoleon called statistical medicine into life and bade it tell him how many men, fit for soldiers, a given population, in a given time, would be able to send to his camp. The answer not being satisfactory, for the want of sufficient data applicable to France, he set about to collect further statistical details. The contributions to this department of our science, under his orders, are highly valuable, in consequence of the great pains taken to guard against errors in the data. To guard against errors from immigration and emigration, he had a cordon of soldiers stretched around a number of the departments of France, containing a population of some three millions of souls. All the inhabitants of the district, under observation, were carefully numbered, and due note taken of their age and sex. After three years of observation the facts were generalized, and the interrogatory of Napoleon was answered by statistical medicine in a satisfactory manner. The mortality was found to be about one in thirty. That is, one individual died every year among every thirty persons; but more than one individual was born every year among every thirty persons; and thus the ratio of increase over the decrease, was ascertained. It is contrary to the order of nature, that population should decrease, unless impeded by some evident local cause. That want of territorial space does not prevent population from increasing, in any country not more populous than France, is proved by the facts just mentioned.

Having alluded to Napoleon's contributions to statistical medicine, I have now to allude to more valuable contributions to that department of our science from a greater man than he

—our own glorious Washington. Early in the first term of Washington's administration, provision was made for the collection of facts, of the most valuable kind to statistical medicine, and immediately carried into effect under his authority. That collection of statistical details, made in 1790, formed a precedent, which has been followed every ten years since that period with a similar collection, under the modest title of "Census." Six have already been taken, and it will soon be time for the seventh. An immense amount of valuable statistical matter has been collected together from every county and sub-division of a county, from every village, town or city, and from every ward of every city, in the whole Union, and published by acts of Congress, forming six very voluminous works, under the unpretending title of "*Census.*" The title does not suggest to the mind, at first sight, any connexion with the medical science. Yet on a little consideration, and particularly by casting the eye over the contents of those massive volumes, (containing the largest amount of authentic statistical details that any country possesses,) it will at once appear to every reflecting mind, that they constitute a sufficient foundation for building up a noble superstructure of American Statistical Medicine, Washington being its founder, as he is also the founder of our political liberties. The same great man, who, aided by his compatriots and the wise men of the age in which he lived, as Franklin, Jefferson, Madison, Hamilton, Adams, Henry, and others, broke up quackery in government, and the artificial distinctions in society, which nourished and fed the political empiricism and founded a new system of government based upon natural distinctions in society and the eternal truths of the Baconian philosophy, (which Hypocrates first taught,) was the very man who laid the foundation of a system of American statistical medicine, destined, ere long, to expel quackery in medicine from the length and breadth of this broad land. The work of Washington in freeing his countrymen from the evils of knavery and empiricism in the science of government, would only have been half complete if his wisdom and foresight had not devised some means by which they could, in due course of time, be freed from the evils of knavery and empiricism in medicine. Those who do not see how this is to be effected should recollect, that the practice of the medical men attached to the Prussian, French and British armies and navies, is tested with great accuracy from data similar to those furnished by the United States Census. The numerical method, the data being given, discloses the number who die between certain ages, and thus, the relative mortality can be ascertained in each separate locality, and the different localities compared with one another will show, by their greater or less ratio of mortality, (other things being equal,) the blessings of the healing art and the evils of ignorance, imposition and quackery in medicine. I have paid some attention to this subject and have made many calculations to find out the localities where the mortality is the highest and the lowest, and thus be able to trace the location of empiricism and imposition in medicine on the map of the United States.

The manuscript containing the results of my investigation I left at home, in Natchez. I can, therefore, only point out a few of the general conclusions arrived at, which I have retained in memory. Any one, who will take the trouble to make the calculations from the data afforded by the census, must, from necessity, arrive at the same conclusions. Hence statistical medicine is often called political arithmetic. One of the general conclusions demonstrated by American political arithmetic is the most important one, that human life in the Mississippi valley, taken as a whole, is not as safe as human life east of the Alleghany mountains. Thus, it will be found, that out of the whole number of inhabitants between twenty and thirty years of age, a fewer number reach forty, fifty, sixty or seventy years of age in the west than in the east. There is also a great difference in the duration of life in different states of the valley and in different counties in the states, and a difference in different townships or precincts of the same county. Thus, the term of human life is longer in Kentucky than in Ohio—longer in Ohio than in Indiana; and longer in

Indiana than in Illinois. Three great causes operate to shorten human existence in this valley—one is malaria and the too common practice of treating malarious or bilious affections, congestive fever, pneumonia, &c., as the diseases of hyperborean Europe are treated. When the long promised work of Dr. Drake on the climate, diseases, and medical geography of the Mississippi valley makes its appearance, it will, no doubt, demonstrate the error of making the writings of hyperborean physicians our guide in treating a formidable class of hot-weather fevers, unknown in the cold climates, where those authors reside; and thus lead to a better mode of practice by making us independent of hyperborean Europe for books, in the treatment of our endemial diseases. I am sanguine in the belief that malarial diseases, not only admit of a better mode of treatment, but that they may, in a great measure be prevented, and that when the malarial cause cannot be removed, its injurious effects on the system may be, in a great degree, counteracted.

The second cause of the great mortality in this valley is the large amount of adulterated stale and worthless medicine sent here for sale, which will sell no where else. Physicians cannot expect to be successful unless they have good medicine. The too common practice of our apothecaries of purchasing their drugs from wholesale drug establishments, whose proprietors are also the proprietors of nostrums and patent medicines of their own, and who are interested in bringing the regular articles of the materia medica into disrepute, should be broken up, or our physicians should take the management of the materia medica back into their own hands and dispense with the agency of apothecaries entirely.

The third and main cause of the great mortality in this valley, besides what is caused by the direct agency of ignorant and empirical practitioners, is owing to the immense consumption of nostrums and patent medicines of all kinds, by our western people in attempting to doctor themselves. From finding among the patented medicines, occasionally a good vermifuge or a good aperient in those pills which are made of socotorine aloes, or a good remedy for intermittent fever in those nostrums containing genuine sulphate of quinine, the confidence of the people is often so far *entrapped*, as to make a hap-hazzard use of quack nostrums for all other complaints.

The great and unnecessary sacrifice of human life, from such causes, is not seen so clearly owing to the rapid increase of the population.

On recurring to those valuable statistical details, which we owe to the foresight of Washington, we will find from the data there afforded us, why the population in the west increases so fast, notwithstanding the high ratio of mortality. The numerical method, applied to the data there afforded us, will demonstrate, with mathematical certainty, that the wonderful increase of the population is owing to the women in the Mississippi valley being twice as *prolific*, or nearly so, as the women east of the Alleghany mountains. Thus it will be found, on examination, that for every 1000 women between fifteen and fifty, east of the mountains, there is only on an average about 1200 children, or persons under 15 years of age: Whereas for every 1000 women between fifteen and fifty, west of the mountains, there is on an average about 2200 children or persons under fifteen years of age.

The women in the Mississippi valley are therefore nearly twice as prolific as those in the Atlantic States. If there were no emigration from east to west and the mortality was the same, the west would double its population in nearly half the time of the east. But it does not do this, when we take into consideration the great number it gains per annum and the east loses, by removals from east to west. What it lacks in doing so, is lost by an increase of mortality. As there are 2200 children for every 1000 females west, and 1200 children for every 1000 females east, and as the number of males is nearly the same with the females, it follows that the whole population between fifteen and fifty, should double itself in about thirteen years and a half in the west, and in twenty-five years in the east from natural causes, provided the mortality was the same in each, and there was no emigration. I have not time to dwell on this subject, but I will say to the young gentlemen of the University, that if they want to find healthy or sickly neighborhoods in which to locate themselves in the practice of medicine, they have only to get the full returns contained in those large works called the *Census*, and find out by the special returns from each county and sub-division of a county. or from each city and sub-division of each city into wards, the proportion of the population under, or between certain ages, and then by calculations and comparisons they can ascertain the most healthy or sickly locality in any part of the United States with almost positive certainty. There is, however, not always the most sickness to be found in places the most obnoxious to life. It is generally the reverse—there being more cures of disease in the aggregate in a salubrious than in a noxious atmosphere. In the latter, deaths are numerous and maladies short in their progress. In the former, though the deaths be few, the indispositions are numerous, and often of long continuance. Morbidity and mortality are often in an inverse ratio. Knowledge is power—power even to choose a good location—a most important matter to a young physician in setting out in life.

The proprietors and venders of nostrums and quack preparations, not content with the profits derived from the impositions practiced on the male part of the community, derive great gains from similar impositions practiced on females, under pretence of curing their particular diseases and preserving their health and beauty. The various dentifrices for the teeth, preparations to prevent the hair from falling out—cosmetics to improve the complexion—lotions to soften and whiten the skin and to remove pimples and other cutaneous blemishes; and numerous nostrums for chronic uterine affections from being used indiscriminately, without regard to the circumstances of the constitution, the general health and particular temperament, not only fail, in most instances, but injure the health and spoil the very beauty they promise to improve or preserve. Under quack dentifrices to make the teeth look pretty and white the enamel is often destroyed and the teeth are lost entirely. Not knowing that the hair is subject to a variety of diseases, requiring local and constitutional treatment, differing to suit the particular case, the empirical remedies, used indiscriminately, are apt to make the hair fall out or to become rough and uncouth, as to make it grow and become soft and glossy. The Grecian women sacrificed the locks of their hair to Hygeia, the goddess of Health, and daughter of Æsculapius. This practice is allegorical of the necessity of improving the health in order to preserve the hair. There are many diseases of the viscera, which thicken, pimple, blotch or discolor the skin, and consequently many of the lotions and other preparations professing to soften and whiten the complexion, are apt to kindle up some internal disease of the lungs or other viscera. In regard to the astringent and other quack nostrums, so extensively used in the uterine profluvia, they seldom or ever cure the disease. If they succeed in arresting a discharge, without removing the cause, the disease of the uterus is translated to the head, stomach, heart, liver, skin, or nervous system, and not cured. A thousand beautiful complexions are ruined by such remedies. If the disease is not translated, the uterine tissues thicken, when the profluvia, which generally depends on some inflammatory action or irritation, causing a fluxionary movement to the organ, are suddenly arrested by astringents. In consequence of this thickening or engorgement of the uterine tissues, the organ becomes of more than twice its natural weight, and prolapsus is the necessary consequence of this increase, which nothing will cure that does not remove the engorgement, and reduce the organ to its natural size. Pessaries and supporters are only miserable palliatives of a disease, requiring a high exercise of professional skill to prevent or to remove after it has occurred.

Who so capable of improving a complexion and of curing the many diseases to which the skin and the uterus, with which it sympathises, are so subject, as the physician, who has studied their anatomy, physiology and pathology, and the action and reaction of sympathetic influences, and whose eye can see through those very blemishes and discolorations of the skin, as through a glass, into the derangement of viscera of which they are often the indexes? Who so well capable of giving advice to preserve the teeth, as he who knows the origin of the various diseases of the gums and the general constitutional causes, which make them decay, in defiance of quack dentifrices? About half the decayed teeth in this valley may be fairly attributed to a scorbutic or impoverished condition of the circulating fluids, which is the effect of previous disease, or the effect of an improper diet, or what is most common, the effect of breathing a malarious atmosphere—not to be cured by dentifrices—but by a proper diet,—or by a course of anti-scorbutic remedies, or by tonics to strengthen the system.

Nature has been more kind to the American women than to any other portion of the daughters of Eve. To preserve their health and beauty, American physicians have done little or nothing. With the exception of the maladies common to the two sexes, and a few acute diseases, peculiar to females, the American women may be said to have no physician; because for every chronic ailment peculiar to them, which does not absolutely confine them to their room or bed, but which nevertheless undermines their constitution, spoils their beauty and makes them look old before they have reached the meridian of life, they generally give themselves over to empirics of their own or our sex, or undertake to treat themselves by nostrums and quack medicines, or else they let the worm, at the bud of youth, health and beauty, gnaw on and complete its ravages unmolested.

Statistical medicine proves that American women grow old, lose their youthful beauty and perish at an earlier period of life than the women of other civilized countries. There is a cause for this. The medical profession in this country has too much neglected the minor affections and the more slow and insidious ailments peculiar to females, and they, on their part,

2*

are too reserved, distant and shy of the profession to consult it, when not driven to do so by danger or extreme suffering. In fact the great portion of them look upon the faculty as spoilers rather than preservers of health and beauty. This unfavorable opinion has its origin in unnecessary salivations and the neglect, after an acute disease has run its course, to give the necessary advice to repair the ravages the complaint has made, and to re-establish the health, as perfect as it was before, by a proper diet and regimen. When life is safe and convalescence begins, both patient and physician are too apt to fall into the error, that there is nothing more for science to do. The same science which saved life, in an active disease, is often still required to repair any damages the disease may have inflicted on the constitution. Unless this be attended to, the patient is apt to fall into the too common error of supposing, that it was the remedies employed, and not the disease, which injured the constitution. It was never intended that the diseases of females should be left to empirics of either sex, or that they should doctor themselves. By a most wonderful and beautiful provision of nature, as if to save delicacy of feeling, and to do away with the necessity of the first revelation by words coming from them, Nature has stamped the characters of the diseases peculiar to the sex, in their countenance and expression, which none can read but the scientific physician who has deeply studied their complaints. Reading the disease in their countenance, he can generally tell what they complain of, and perceiving that he knows without telling, will be less disinclined to submit to the necessary examinations to ascertain its progress and its particular location. That branch of science called *prosoposcopia*, or the science of detecting disease by the expression of the countenance is very serviceable in the treatment of the diseases of women and children. The latter cannot, and the former often will not, from mere bashfulness, tell what is the matter with them. Diseases, like the passions, have their peculiar expressions—particularly the uterine affections. There is always something in the expression, look, gait, complexion or attitude, to enable the observant physician to detect such affections at a glance.

By studying a little more attentively this branch of our science, the faculty will greatly benefit the fair sex and at the same time starve out quackery, as fully a moiety of those ill got gains, which feed and fatten empiricism, is derived from the sale of an immense amount of nostrums, fabricated for female consumption, and which answer no other purpose than to commit depredations on their constitutions, spoil their beauty, and swell the bills of mortality.

Statistical medicine is not confined solely to a registration of births and deaths. It registers all unsuccessful plans of treating disease, and the false theories on which such plans of treatment are founded. The most of medical theories are true to a certain extent. But the moment the theory is stretched beyond the narrow sphere of the facts on which it is built, it becomes a false and dangerous guide in medicine. From the prevalence, a few centuries ago, of a fever called putrid, a class of remedies, called antiseptics, came into vogue. They were found to be useful in the complaint then prevalent. This fact gave rise to the theory, that the diseases then prevailing, were owing to a septic tendency of the humors. But soon the theory was stretched beyond the sphere of the facts on which it was founded, and all fevers, and all diseases, even inflammation itself, were supposed to arise from one and the same cause. Hence antiseptics became the reigning hobby for the cure of all diseases. The practice, under the theory, failed of success. But the want of success was attributed to the want of some more powerful antiseptic, and not to the want of truth in the theory. Supposing that the most powerful antiseptics were known to the Egyptians and used by them in preserving the human body from decay, the catacombs of Egypt were ransacked, and the mummies were brought to Europe, and manufactured into medicine, and given as the universal physic. Soon that terrible enemy of quackery and false doctrines, statistical medicine, declared against the mummy practice. It broke into fragments, and was reformed by mixing other substances with the mummy. One branch of the reformed practice, particularly in diseases of the biliary organs, was proved by statistical medicine to succeed admirably. The medicine which answered so good a purpose was called mummy of mercury. This medicine has come down to us, but we no longer call it by the name it first bore—we call it *calomel.* This antiseptic theory should be a caution against using any one remedy, or a little class of remedies, in all diseases indiscriminately.

Steaming is very good in its place. I had often steamed patients before I ever heard of Thompson, the steam-doctor. But when we apply steam in all cases, we fall into the same errors, as those who gave antiseptics in all cases—or bleed and give calomel in all cases—or Cooke's pills in all cases—or gum water in all cases. I am fond of roots and herbs from our woods, and had long used them before I ever heard of Botanical physicians. But I should be very sorry to be tied down by any theory to the exclusive use of roots and herbs. I have seen the lancet do good and save life in many inflammatory diseases. I would think it morally wrong to stand by and see a patient die in all the agonies of an acute inflammation, when timely bloodletting would save him, and let my hands be tied by a theory against bloodletting founded on its abuses. I have seen lobelia and red pepper abused. But I have not been deterred by such abuses from using those articles in cases where the experience of the world before me and the world around me, added to my own, had proved them to be the best remedies. I learned the virtues of red pepper from the ancients, and from our West India practitioners. The virtues of lobelia I have picked up from a variety of sources—steam doctors among the rest.

We all know, from the truths revealed by statistical medicine, that physicians in all ages and countries, as a general rule, are not well qualified to prescribe for themselves.—When they do prescribe for themselves they are more apt to die than when they are prescribed for by other physicians. Dr. Rush died in consequence of undertaking to prescribe for himself. Yet in the face of this important fact, we see catchpenny books, ever and anon, issued from the press, leading the people to believe that every man can be his own doctor, and every *woman too!* For instance, there is just published a work called 'the Married Woman's Private Companion,' intended to make women believe that each one can cure herself, if she will read that work, and take the patent medicine therein recommended, at five and ten dollars a box. There is not a word in the book which is true that physicians did not know, and nothing in it that it would do females any good to know. Besides it is calculated to frighten them by dwelling on Cæsarian operations, and mal-formations, as if they were common occurrences. It is calculated to mislead them into the belief, that from the anatomy and physiology it reveals, with the help of the patent medicines and a few simples, they can become their own doctors. If the fact were known, that the ablest physicians, with all the experience and lights of science around them, cannot safely doctor themselves, how plain would it appear that no woman can safely doctor herself by such a book and the remedies therein advised. The diseases it proposes to cure so readily, and the anatomy and physiology it proposes to teach so easily, are the most difficult subjects in the science of medicine. This, however, is only one of the numerous works calculated to deceive and mislead the public into the fatal error, that man, as well as woman, can be their own doctor. Physicians have done very little to dispel this delusion. It is a delusion which greatly increases the mortality in this valley. Whether they ought to make any attempt or not to enlighten the public mind on a subject of such importance, I do not pretend to decide.

My object is accomplished, gentlemen, in calling your attention to the subject of statistical medicine and in reminding you that you have a light in that department of our science, sufficient to dispel the gross darkness which broods over the public mind on everything connected with the healing art, if you choose to uncover it.

